# Copper and Zinc Treatments Alter the Thyroid Endocrine System in Zebrafish Embryos/Larvae

**DOI:** 10.3390/toxics10120756

**Published:** 2022-12-04

**Authors:** Liqiao Zhong, He Zhang, Luyin Wu, Huijun Ru, Nian Wei, Fan Yao, Zhaohui Ni, Xinbin Duan, Yunfeng Li

**Affiliations:** 1 Fishery Resources and Environmental Science Experimental Station of the Upper-Middle Reaches of Yangtze River (Ministry of Agriculture and Rural Affairs), Yangtze River Fisheries Research Institute, Chinese Academy of Fishery Sciences, Wuhan 430223, China; 2 Zhejiang Provincial Key Lab for Subtropical Water Environment and Marine Biological Resources Protection, College of Life and Environmental Science, Wenzhou University, Wenzhou 325035, China; 3 State Key Laboratory of Biocatalysis and Enzyme Engineering, Hubei Universtiy, Wuhan 430062, China

**Keywords:** copper and zinc, hypothalamus-pituitary-thyroid axis, thyroid disruption, zebrafish

## Abstract

Copper (Cu^2+^) and zinc (Zn^2+^) are two kinds of heavy metals essential to living organisms. Cu^2+^ and Zn^2+^ at excessive concentrations can cause adverse effects on animals, but little is known about the thyroid-disrupting effects of these metals in fish, especially in the early developmental transition stage from embryos to larvae. Wild-type zebrafish embryos were used to expose to Cu^2+^ (0, 1.5, 15, and 150 μg/L) and Zn^2+^ (0, 20, 200, and 2000 μg/L) for 120 h. Thyroid hormone contents and transcriptional changes of the genes connected with the hypothalamic-pituitary-thyroid (HPT) axis were measured. Results showed that zebrafish embryos/larvae malformation rates were significantly increased in the Cu^2+^ and Zn^2+^ groups. Remarkably elevated thyroxine (T4) concentrations and reduced triiodothyronine (T3) concentrations were observed in Cu^2+^ and Zn^2+^ exposure fish. And the expression patterns of genes connected with the HPT axis were changed after Cu^2+^ and Zn^2+^ treatment. Based on principal component analysis (PCA) results, Zn^2+^ caused significant effects on the thyroid endocrine system at 200 μg/L, while Cu^2+^ resulted in thyroid disruption as low as 1.5 μg/L. In short, our study demonstrated that exposure to Cu^2+^ and Zn^2+^ induced developmental toxicity and thyroid disruption to zebrafish embryos/larvae.

## 1. Introduction

Heavy metals are defined as elements having atomic numbers larger than 20 and atomic densities larger than 5 g/cm [1,2]. They are produced from natural and human activities [3,4]. Heavy metals are not biodegradable or chemically degradable but can be bioaccumulated through the food chain in organisms [5,6]. These heavy metal ions enter the aquatic ecosystem through different channels such as geological weathering, atmospheric precipitation, and discharge of industrial or agricultural waste products [3]. They can be classified as essential and nonessential heavy metals [1,7]. Copper (Cu^2+^) and zinc (Zn^2+^) are two trace elements essential to living organisms [8,9,10]; they are important for the activities of multiple enzymes and play a significant role in a variety of biochemical responses [9,11]. However, when the concentrations increase, these essential heavy metals can induce harmful effects on organisms [4].

The toxicity of Cu^2+^ has attracted significant attention. Exposure to Cu^2+^ resulted in developmental toxicity, DNA damage, oxidative stress, hepatotoxicity and neurotoxicity in aquatic organisms [4,12,13,14,15]. However, little attention has been paid to the effects of Cu^2+^ on the endocrine system, especially the thyroid endocrine system. Previous studies have shown that copper deficiency could induce an increase in serum triiodothyronine (T3) levels in male Sprague-Dawley rats [16]. Similarly, Cu^2+^ deficiency in pregnant rats significantly reduced serum total thyroxine (T4) and T3 levels in neonatal rats [17]. In fish, exposure to Cu^2+^ for 7 days significantly elevated thyroid hormones (THs, including T4 and T3) in three-spined sticklebacks [18]. The THs were altered when rainbow trout and common carp were exposed to Cu^2+^ at the initial stage [19].

The effects of Zn^2+^ have been well documented [20]. Zinc deficiency could influence DNA repair and antioxidant defenses in rats [21], while exposure to excessive Zn^2+^ caused developmental toxicity in zebrafish and rare minnow [22,23]. The toxicity mechanisms of Zn^2+^ include disrupting calcium absorption, inhibiting Na^+^/K^+^-ATPase, inducing oxidative stress, and so on [24]. However, relatively little attention has been paid to the adverse effects of Zn^2+^ on the endocrine system. Zn^2+^ is an essential trace element for normal contents of T3, T4 and thyroid-stimulating hormones (TSH) [25]. Previous studies reported that Zn^2+^ was positively correlated with serum TSH levels in patients with hyperthyroidism [26]. The extrathyroidal production of T3 was impaired in zinc-deficient male Sprague-Dawley rats [27]. Experimental investigation showed that Zn^2+^ played an important role in THS metabolism through affecting deiodinase (Dio) enzyme activity and regulating the synthesis of TSH and thyrotropin-releasing hormones (TRH) [28]. However, the relationship between Zn^2+^ and the thyroid endocrine system in fish is unclear.

THs, which are synthesized in the thyroid gland, play crucial roles in multiple biological processes in vertebrates, such as growth, differentiation, metabolism, nervous system development, and reproduction [29,30,31,32]. Thyroid functions are predominantly regulated by the hypothalamus-pituitary-thyroid (HPT) axis. The HPT axis primarily controls the THs synthesis, transport and metabolism [33,34].

Although several studies reported that treatment with Cu^2+^ or Zn^2+^ affected THs levels in animals, the data involving thyroid disruption treatment in fish with these essential metals is insufficient, particularly in the early developmental transition stage from embryos to larvae. In this report, zebrafish embryos were used to demonstrate the effects of Cu^2+^ and Zn^2+^ treatment on the thyroid endocrine system. These effects were observed by measuring the levels of THs and gene expression involved in the HPT axis.

## 2. Experimental Procedures

### 2.1. Embryo Culture and Exposure

The zebrafish embryos (AB strain) were procured from the China Zebrafish Resource Center (Wuhan, China). Experimental procedures on fish were approved by the Animal Experimental Ethical Inspection of Laboratory Animal Centre, Yangtze River Fisheries Research Institute, Chinese Academy of Fishery Sciences (No. 20180504001). The toxicological experiments were carried out according to the Chinese national standard: toxicity tests for chemicals using fish in the early-life stage (GB/T 21854-2008). The Holt buffer (composition of: 3.5 g/L NaCl, 0.05 g/L KCl, 0.025 g/L NaHCO_3_, 0.1 g/L CaCl_2_, pH = 7.0) was used to culture the embryos. The cultured conditions that were set were as follows: constant temperatures (28 ± 0.5 °C) with a 14:10 (light: dark) photoperiod. Furthermore, Cu^2+^ and Zn^2+^ stock solutions were prepared after dissolving copper sulfate (CuSO_4_·5H_2_O, CAS: 7758-99-8, purity ≥99.5%, Shanghai Biochemical Technology Co., Ltd, Shanghai, China) and zinc sulfate (ZnSO_4_·7H_2_O, CAS: 7446-20-0; purity ≥99.5%, Shanghai Biochemical Technology Co., Ltd.) in UP water. The embryos were exposed to varying exposure concentrations of Cu^2+^ (0, 1.5, 15, and 150 μg/L), and Zn^2+^ (0, 20, 200, and 2000 μg/L). Healthy embryos (14,700) were randomly assigned to 42 glass beakers (350 per beaker) containing 350 mL exposure solution. Each treatment was conducted in six replicates. These embryos were exposed to the toxic ions for 120 h (5 days), and the exposure solution was replaced by a new solution every 24 h to maintain the Cu^2+^ and Zn^2+^ concentrations. Zebrafish embryos at 0–5 dpf do not need to be fed. Therefore, the embryos were not fed during the exposure period of 120 h. The water samples were collected before renewal of the exposure medium for measuring the actual Cu^2+^ and Zn^2+^ concentrations. The concentrations for these heavy meals were determined by atomic absorption spectrometry according to the method of National Standard of China (GB7475-87). The actual Cu^2+^ and Zn^2+^ concentrations exceeded 80% of the corresponding specific concentrations in all water samples. During the period of exposure, the dead embryos were discarded every day, and the hatching, survival, and malformation rates were recorded at 120 h. The larvae (120 h) were anesthetized with tricaine methanesulfonate (MS-222, Sigma-Aldrich, Saint Louis, MO, USA), randomly sampled for subsequent assays of THs and gene expression levels, and immediately stored at −80 °C.

### 2.2. RNA Extraction and Quantitative RT-PCR

Twenty larvae were selected randomly from every beaker and pooled into a single sample for gene expression analysis. Every group contained six replicates. The total RNA was extracted with the aid of the Trizol reagent (Invitrogen, Carlsbad, CA, USA), based on the manufacturer’s protocols. Agarose gel (1%) electrophoresis was used to estimate the quality of the total extracted RNA. The RNA contents were measured using the Nanodrop lite spectrophotometer (Thermo Fisher, Waltham, MA, USA). FastKing gDNA Dispelling RT SuperMix (Tiangen Biochemical Technology, Beijing, China) was used to synthesize first-strand cDNA, following the manufacturer’s protocols. Quantitative RT-PCR was carried out using the ABI 7500 System (Applied Biosystems 7500, Carlsbad, CA, USA) with the UltraSYBR mixture (Low ROX) (CWBIO Beijing, China), following the kit instructions. Thermal cycling was done at 95 °C for 10 min, followed by 40 cycles of 95 °C for 15 s and 60 °C for 60 s. 18sRNA was selected as a reference gene when calculating the gene expression levels. The 2^-ΔΔCt^ technique was used for analyzing the differences (variations) in the gene expression levels [35]. The quantitative RT-PCR primers were obtained from a previous study [36].

### 2.3. Thyroid Hormone Assays

The whole-body T4 and T3 contents in the larvae were estimated using a previously reported method [37]. Briefly, approximately 200 larvae from each replicate were homogenized with ice-cold phosphate-buffered saline (PBS) with a glass grinder, and the samples were sonicated on ice. Every group included 6 replicates. The supernatants were obtained after the homogenate samples were centrifuged at 5000 rpm at 4 °C for 15 min. The total protein contents in the supernatants were quantified by Bradford assay [38]. The THs (T4 and T3) concentrations from supernatants were estimated using the enzyme-linked immunosorbent assay (ELISA) kit (Cloud-Clone Corp. Wuhan, China, T3: CEA453Ge; T4: CEA452Ge), following kit instructions. The T3 and T4 detection limits were calculated to be 51.7 pg/mL and 1.29 ng/mL, respectively.

### 2.4. Statistical Analysis

The data related to THs contents and gene expression levels were presented as the mean ± standard deviation (SD). The data derived in the study were analyzed with SPSS 20.0 (IBM, Chicago, CA, USA). The Kolmogorov–Smirnov test was employed for validating the data normality. The Levene test was utilized for analyzing the homogeneity of variances. After validating the data normality, the statistical differences observed in the treatment groups were confirmed by means of one-way analysis of variance (ANOVA), followed by Tukey-HSD tests. Principal Component Analysis (PCA) was carried out using Origin 2021 (OriginLab, Northampton, MA, USA). The correlation analysis was confirmed by Spearman’s test. Values with *p* < 0.05 were established as statistically significant.

## 3. Results

### 3.1. Developmental Toxicity Caused by Cu^2+^ and Zn^2+^

The developmental toxicity exposed to Cu^2+^ and Zn^2+^ was illustrated in Table 1. It was seen that the hatching and survival rates were not altered when zebrafish embryos/larvae were exposed to Cu^2+^, while the malformation rate was significantly increased in the 150 μg/L Cu^2+^ group. Similar to Cu^2+^, Zn^2+^ treatment did not alter survival and hatching rates of zebrafish embryos/larvae. However, the rate of malformation was significantly elevated in the 2000 μg/L Zn^2+^ group compared with the control.

### 3.2. Influences of Cu^2+^ on Thyroid Endocrine System

**Figure 1:**
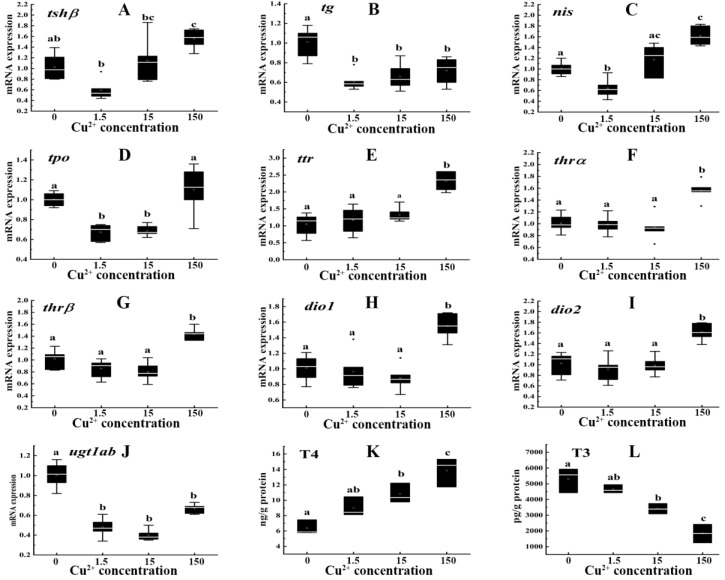
Transcription levels of *tshβ* (**A**), *tg* (**B**), *nis* (**C**), *tpo* (**D**), *ttr* (**E**), *thrα* (**F**), *thrβ* (**G**), *dio1* (**H**), *dio2* (**I**), *ugt1ab* (**J**) and contents of T4 (**K**), T3 (**L**) in zebrafish embryos/larvae that were treated with Cu^2+^ (0, 1.5, 15, and 150 μg/L) for 120 h. Data are shown as mean ± SD (*n* = 6). Different letters denote the significant differences between groups.

Exposure to 150 μg/L Cu^2+^ remarkably upregulated the levels of *tshβ* gene expression (1.53-fold) compared with the control (Figure 1A). The gene expression of thyroglobulin (*tg*) was significantly downregulated 0.60-, 0.65- and 0.71-fold in the 1.5, 15 and 150 μg/L Cu^2+^ exposure groups, respectively (Figure 1B). Expression of the sodium-iodide symporter (*nis*) gene was significantly downregulated 0.63-fold in the 1.5 μg/L Cu^2+^ exposure group but was upregulated 1.60-fold in the 150 μg/L Cu^2+^ exposure group (Figure 1C). Thyroid peroxidase (*tpo*) expression was remarkably downregulated 0.67- and 0.69-fold in the 1.5 and 15 μg/L Cu^2+^ treatment groups, respectively (Figure 1D). The gene expression levels of transthyretin (*ttr*), thyroid hormone receptor-α (*thrα*), thyroid hormone receptor-β (*thrβ*), type I iodothyronine deiodinase (*dio1*) and type II iodothyronine deiodinase (*dio2*) were significantly upregulated 2.22-, 1.54-, 1.43-, 1.55- and 1.58-fold in the 150 μg/L Cu^2+^ treatment group, respectively (Figure 1E–I). However, expression of the uridine diphosphate glucuronosyltransferase 1 family a, b (*ugt1ab*) gene was significantly downregulated 0.48-, 0.39- and 0.66-fold in the 1.5, 15 and 150 μg/L Cu^2+^ exposure groups, respectively (Figure 1J).

Cu^2+^ exposure significantly increased the T4 contents by 1.69- and 2.18- fold in the 15 and 150 μg/L Cu^2+^ treatment groups, respectively (Figure 1K), while the T3 contents were significantly decreased 0.64- and 0.35-fold in the 15 and 150 μg/L Cu^2+^ treatment groups, respectively (Figure 1L).

### 3.3. Influences of Zn^2+^ on Thyroid Endocrine System

**Figure 2:**
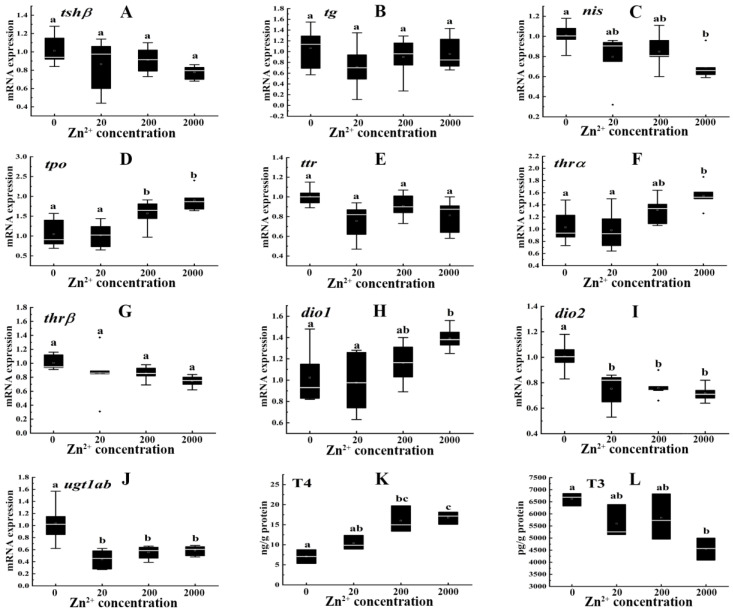
Transcription levels of *tshβ* (**A**), *tg* (**B**), *nis* (**C**), *tpo* (**D**), *ttr* (**E**), *thrα* (**F**), *thrβ* (**G**), *dio1* (**H**), *dio2* (**I**), *ugt1ab* (**J**) and contents of T4 (**K**), T3 (**L**) in zebrafish embryos/larvae that were treated with Zn^2+^ (0, 20, 200 and 2000 μg/L) for 120 h. Data are shown as mean ± SD (*n* = 6). Different letters denote the significant differences between groups.

Downregulation of *nis* (0.69-fold) was ascertained in the 2000 μg/L Zn^2+^ treatment group compared with the control (Figure 2C). Treatment with 200 and 2000 μg/L Zn^2+^ significantly upregulated the mRNA expression of *tpo* (1.50 and 1.81-fold, respectively) compared to the control (Figure 2D). Treatment with 2000 μg/L Zn^2+^ remarkably upregulated the mRNA expression of *thrα* (1.50-fold) and *dio1* (1.36-fold) (Figure 2F,H). The gene expression levels of *dio2* (0.74-, 0.75, and 0.71-fold) and *ugt1ab* (0.42-, 0.53- and 0.56-fold) were remarkably downregulated in all Zn^2+^ treatment groups (20, 200 and 2000 μg/L, respectively) (Figure 2I,J). Nevertheless, *tshβ*, *tg*, *ttr* and *thrβ* expression showed no significant changes (Figure 2A,B,E,G).

Zn^2+^ treatment remarkably increased the T4 content by 2.27- and 2.38-fold in the 200 and 2000 μg/L Zn^2+^ groups, respectively (Figure 2K), while the T3 content was significantly reduced 0.69-fold in the 2000 μg/L Zn^2+^ exposure group compared with the control (Figure 2L).

### 3.4. PCA and Correlation Analysis

**Figure 3:**
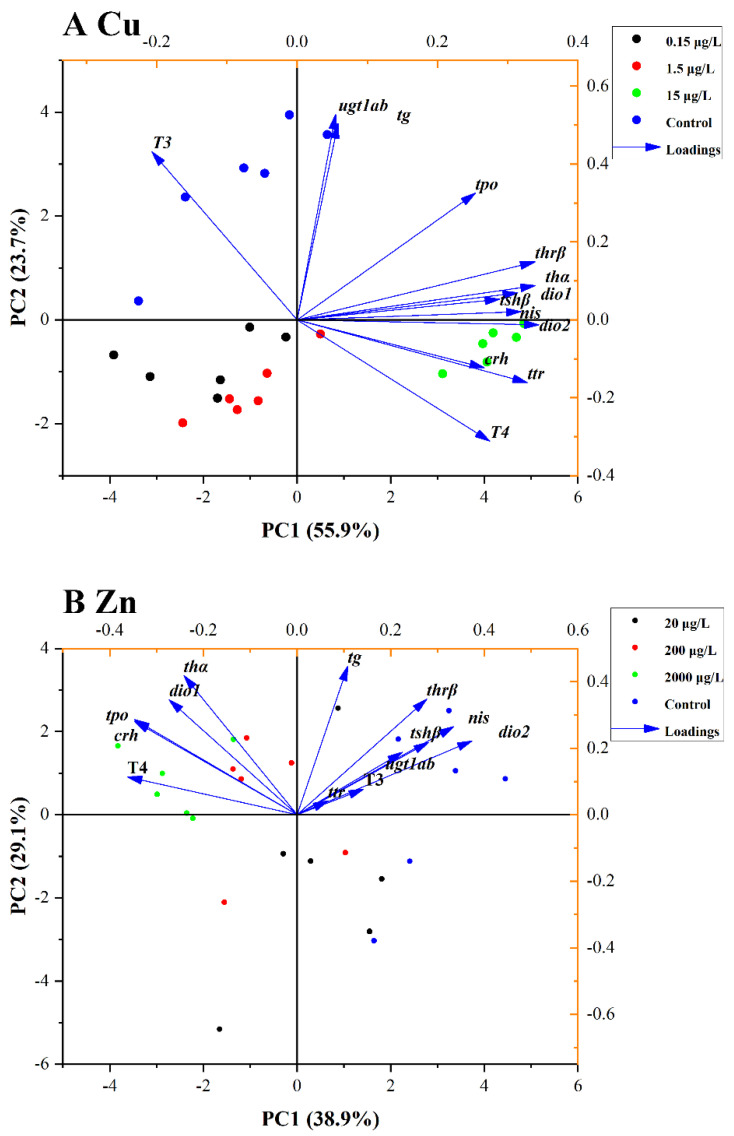
PCA of parameters in zebrafish embryos/larvae that were treated with Cu^2+^ (0, 1.5, 15 and 150 μg/L) (**A**) and Zn^2+^ (0, 20, 200 and 2000 μg/L) (**B**) for 120 h.

**Figure 4:**
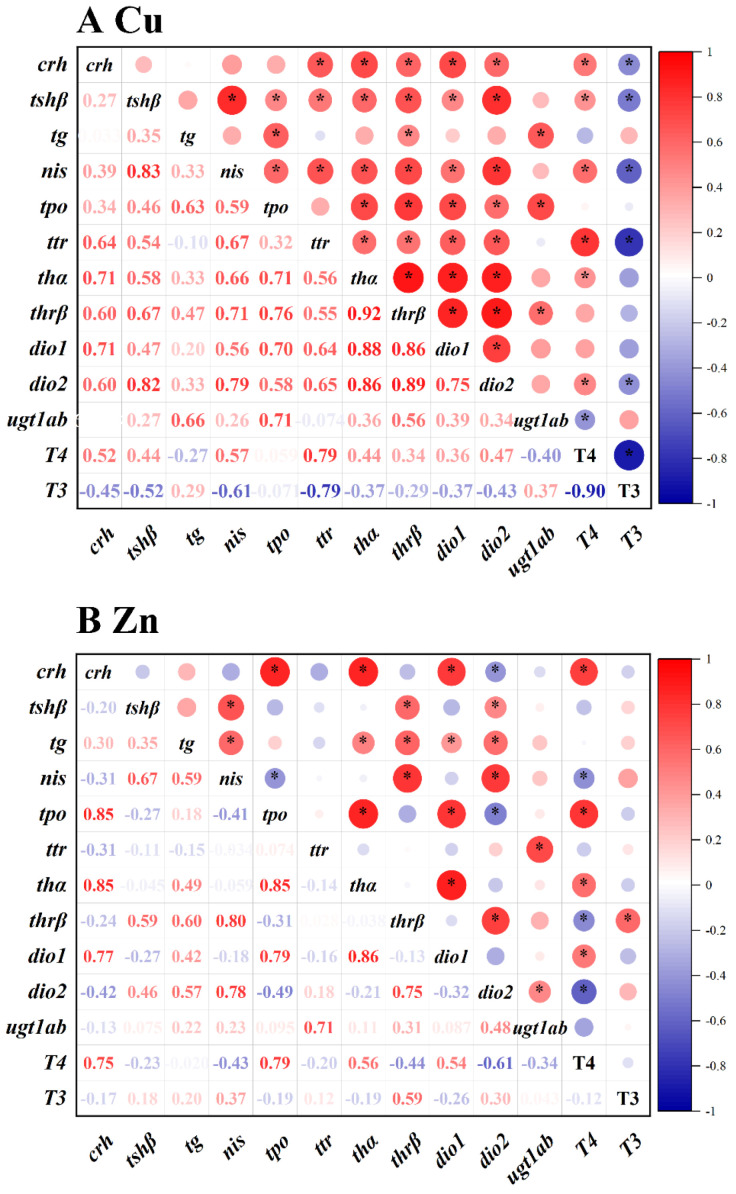
Heatmap of correlation coefficients between the THs (T4 and T3) contents and the gene expression levels in the zebrafish embryos/larvae that were treated with Cu^2+^ (0, 1.5, 15 and 150 μg/L) (**A**) and Zn^2+^ (0, 20, 200 and 2000 μg/L) (**B**) for 120 h. The scale indicates the level of positive (red) or negative (blue) correlation, and * indicates significance (* *p* < 0.05).

Herein, the PCA and correlation analyses were conducted to analyze the relationship between the THs and genes transcriptional levels. The contents of T4 and T3 and transcriptional levels of genes related to HPT axis were used for PCA and correlation analyses. The PCA result of Cu^2+^ exposure is shown in Figure 3A. The initial two principal components (PCs) explained 79.6% of the total variances. The PC1 explained 55.9% of the total variances, while PC2 accounted for 23.7% of the total variances. According to the PCA plot, the separation between clusters of control and 1.5 and 15 μg/L Cu^2+^ groups were significant. T4 level had a strong negative correlation with T3 level; T4 level positively and significantly correlated with the transcriptional levels of *crh*, *tshβ*, *nis*, *ttr*, *thα* and *dio2* and negatively correlated with the transcriptional levels of *ugt1ab* (Figure 4A). However, the T3 level exhibited a strong negative correlation with the expression levels of *crh*, *tshβ*, *nis*, *ttr*, *thα* and *dio2* (Figure 4A).

The PCA result of Zn^2+^ exposure is shown in Figure 3B. The initial two PCs explained 68.0% of the total variances. The PC1 accounted for 38.9% of the total variances, while PC2 explained 29.1% of the total variances. Based on the PCA result, the separation between clusters of control and 200 and 2000 μg/L Zn^2+^ groups were significant. T4 level were seen to be significantly and positively correlated with the transcriptional levels of *crh*, *tpo*, *thα* and *dio1* and were negatively related to the transcriptional levels of *nis*, *thrβ* and *dio2* (Figure 4B), while there was a strong positive correlation between T3 level and *thrβ* transcriptional levels (Figure 4B).

## 4. Discussion

Heavy metals can cause developmental toxicity in fish [14,39]. In the present study, although the hatching and survival rates of the fish were not notably affected, the malformation rates were notably enhanced in the zebrafish larvae exposed to Cu^2+^ and Zn^2+^, particularly in the highest exposure groups (Table 1). Therefore, malformations were found to be a more susceptible parameter compared to hatching and survival rates for assessing developmental toxicity in zebrafish embryos.

In vertebrates, TSH is encoded by the *tshβ* gene, which stimulates the thyroid gland to generate THs via binding to the corresponding receptor [40]. Thus, the upregulation of *tshβ* could be linked to a higher T4 level. In this study, the *tshβ* transcriptional levels were upregulated significantly, which could explain the enhanced T4 concentrations in the Cu^2+^ exposure group. However, although T4 content was significantly increased, the *tshβ* expression showed no significant change in Zn^2+^-treated zebrafish embryos/larvae. Consistent with our results, adult male rat exposure to Zn^2+^ (0 and 3 mg/kg) showed increased free T4 content but no significant changes in TSH content [41]. Therefore, our results indicated that the correlation between TSH content and Zn^2+^ concentration was not strong.

Herein, the expression levels of genes (including *tg*, *nis* and *tpo*) were determined to be associated with THs biosynthesis. The *nis* gene facilitates the iodide accumulation from blood in thyroid cells [42]. The function of the TPO enzyme is to catalyze the iodination of tyrosyl-residues in TG protein and follow-up coupling of iodotyrosines to produce THs [43,44]. Therefore, the upregulation of *tg*, *nis* and *tpo* expressions might be related to the increase of T4 level. In accordance with this, increased T4 contents along with upregulation of *nis* expression and upregulation of *tpo* expressions were observed in the Cu^2+^ and Zn^2+^ groups, respectively. However, the T4 levels were increased, and the *tg* and *nis* genes expressions were significantly downregulated in the Cu^2+^ and Zn^2+^ groups, respectively. This could be attributed to the self-compensatory mechanism in the HPT axis, which downregulated *tg* or *nis* gene expression to decrease the T4 content.

TTR acted as a carrier protein for THs, and it helped in transporting THs to the target tissues through blood circulation [45,46]. Earlier reports demonstrated that T4 content was significantly and positively linked to the *ttr* expression [37,47,48]. In line with these studies, the T4 content was significantly increased, along with upregulated *ttr* gene transcription in Cu^2+^ treatment groups. The THR, which belongs to the nuclear receptor superfamily, is a transcription factor that responds to T3. Previous results showed that T4 bound to *trα* and mediated the expression of THs-regulated genes [49]. Significant upregulation of *thrα* expression was observed after Cu^2+^ and Zn^2+^ exposure in this study, which might be attributed to the higher T4 levels. Previous studies have reported significant downregulation of *thrα* and *thrβ* in Chinese toad following Cu^2+^ exposure [50], while the expression levels of *thrα* and *thrβ* were significantly upregulated in the Cu^2+^ exposure group in this study. These inconsistent consequences might be due to the species-specific effects caused by Cu^2+^ and require further study.

Previous studies reported that Cu^2+^ deficiency and Zn^2+^ supplementation could alter deiodinase activity [28,51]. *Dio2* was involved in T4 deiodination to form active T3 [52,53]. Thus, downregulation of the *dio2* gene expression was attributed to the increased T4 level and reduced T3 level in the Zn^2+^ treatment groups in this study. Moreover, *dio1* functions as an outer-ring THs deiodinase and is involved in the metabolism of THs (including T3 and T4) [54]. Therefore, a decrease in the T3 content in the Zn^2+^ and Cu^2+^ treatment groups was partially attributed to the upregulation of *dio1* expression. Consistent with these results, an increase in the Dio1 activity was reported in Zn^2+^ supplementation rats [55]. The *ugt1ab* gene was remarkably involved in the THs homeostasis by regulating T4 glucuronidation [56]. An earlier study reported that an increase in the T4 content in zebrafish larvae was due to the downregulation of *ugt1ab* mRNA levels [57]. In this study, the *ugt1ab* gene transcription level was significantly downregulated in the Cu^2+^ and Zn^2+^ treatment groups. Therefore, the increased T4 content could be attributed to the downregulation of *ugt1ab* transcription levels.

Based on the PCA results, it was seen that Zn^2+^ significantly affected the thyroid endocrine system at the concentration of 200 μg/L, whereas Cu^2+^ led to thyroid disruption at a concentration as low as 1.5 μg/L. Cu^2+^ was more sensitive compared to Zn^2+^ in the endocrine thyroid system. The Standards for Drinking Water Quality of China (GB5749–2022) state that the maximum allowable concentrations of Zn^2+^ and Cu^2+^ are both 1000 µg/L. Since both Zn^2+^ and Cu^2+^ negatively affected the endocrine thyroid system below this permissible level, the adverse effects of these metals on the endocrine system of fish needs to be investigated further.

## 5. Conclusions

In summary, this study demonstrated that treatment with Zn^2+^ and Cu^2+^ caused developmental toxicity and thyroid disruption in the zebrafish embryos/larvae. Both whole-body THs contents and the expression patterns of genes linked to the HPT axis were altered after exposure to Zn^2+^ and Cu^2+^. Further research is necessary to elucidate the mechanisms of thyroid disruption due to heavy metals toxicity.

## Figures and Tables

**Table 1:** The changes of hatching, malformation, and survival rates in zebrafish embryos/larvae treatment with Cu^2+^ and Zn^2+^. Data are shown as the mean ± SD (*n* = 6). * *p* < 0.05, denotes a statistically significant difference between the treatment and control.

Cu^2+^ (μg/L)	0	1.5	15	150
Hatching (%)	89.83 ± 1.29	88.25 ± 1.32	87.50 ± 2.14	85.58 ± 1.87
Malformation (%)	0.83 ± 0.14	1.92 ± 0.38	2.25 ± 0.66	5.12 ± 0.80 *
Survival (%)	89.25 ± 1.40	87.50 ± 1.40	87.50 ± 2.4	84.83 ± 2.13
Zn^2+^ (μg/L)	0	20	200	2000
Hatching (%)	88.83 ± 1.53	87.08 ± 1.23	87.00 ± 2.29	83.83 ± 1.61
Malformation (%)	1.08 ± 0.38	1.75 ± 0.66	3.5 ± 0.75	4.67 ± 1.13 *
Survival (%)	88.33 ± 1.53	86.25 ± 0.86	85.92 ± 2.47	83.33 ± 1.84

## Data Availability

Not applicable.
